# How I do it: Selective femoral neurotomy

**DOI:** 10.1007/s00701-025-06466-y

**Published:** 2025-03-25

**Authors:** Pierre Petitet, Corentin Dauleac, Camilla de Laurentis, Patrick Mertens

**Affiliations:** 1 Service de Neurochirurgie fonctionnelle, de la moelle spinale et des nerfs périphériques, Hospices Civils de Lyon, Hôpital neurologique et neurochirurgical Pierre Wertheimer, 59 boulevard Pinel, Bron, 69500 France; 2 Université Claude Bernard Lyon 1, Lyon, France

**Keywords:** Selective periphery neurotomy, Spasticity, Stiff knee, Microsurgery

## Abstract

**Background:**

Quadriceps spasticity is responsible for a gait disturbance characterized by stiff knee with reduced knee flexion during the swing phase. Selective Femoral Neurotomy induces long-term muscle relaxation via a decrease of the stretch reflex.

**Method:**

Femoral nerve trunk is dissected just below the femoral crease. Motor branches to the rectus femoris and/or vastus intermedius muscles are identified using electrical stimulation combined with electromyographic recording, then partially sectioned according to an individualized preoperative chart. Postoperative rehabilitation is imperative for sustained gait improvements.

**Conclusion:**

Selective Femoral Neurotomy (SFN) is a lesion-based, permanent surgical treatment of spastic stiff knee gait.

**Supplementary Information:**

The online version contains supplementary material available at 10.1007/s00701-025-06466-y.

## Relevant surgical anatomy

**Fig. 1:**
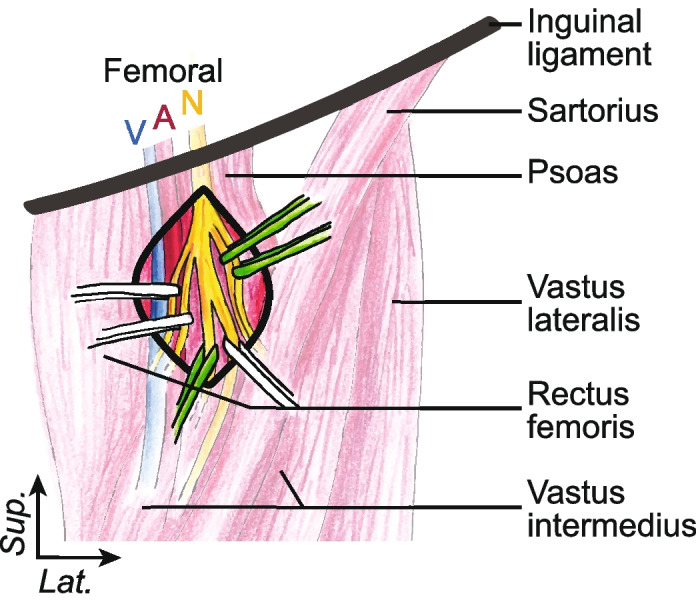
**Relevant surgical anatomy of the left femoral nerve.** V: Vein; A: Artery; N: Nerve

The femoral nerve is approached just below the inguinal crease, after its passing under the inguinal ligament where it becomes lateral to the femoral artery and vein (Fig. 1). Branching of the femoral nerve occurs typically as it enters the thight but in some cases it occurs before it leaves the pelvis [5]. Collateral motor branches are distributed in a fan shape for the rectus femoris superficially, vastus intermedius deeply, vastus medialis medially and vastus lateralis laterally. Collateral sensitive branches of the femoral nerve are the medial and intermediate cutaneous nerve of the thigh and the saphenous nerve.

## Description of the technique

By lesioning motor nerve branches, selective neurotomy aims to locally reduce the hyperexcitability of the myotatic reflex – which is abnormally exaggerated in spastic muscles [8]. Physiologically, this effect primarily stems from disrupting proprioceptive signals of type Ia fibers rather than motor signals of $\alpha$ motor fibers. While the latter sprout post-neurotomy to support motor strength recovery, the former do not, ensuring sustained reduction in reflex hyperexcitability and preventing spasticity recurrence. This phenomenon has been confirmed by electrophysiological studies in human [7]. Below is a step-by-step account of the technique.

- An operative chart describing the percentage of each muscular nerve to be cut is established preoperatively based on clinical evaluation (considering spasticity, muscular strength, consequences of femoral nerve bloc-test with gait analysis for walking patients and individualized patient goals). This process is mostly guided by the modified Ashworth Scale (MAS) as follows: 75% for MAS 4/4, 66% for MAS 3/4, and 50% for MAS 2/4 in quadriceps. Complete neurotomy is never performed to avoid compromising upright posture.
- General anesthesia without curarization is performed, allowing for electromyographic (EMG) monitoring during the procedure.
- The patient is in the supine position with extended legs.
- EMG needle electrodes are placed in each head of the quadriceps muscle for intraoperative mapping of the motor branches (in vastus medialis, rectus femoris and vastus lateralis). Note that the vastus intermedialis muscle is located deeper in the thigh compared to the other heads, making its intraoperative EMG monitoring more difficult.
- Doppler ultrasonography can be used to localize the femoral artery, the femoral vein and the femoral nerve. The femoral nerve is typically lateral to the artery (Fig. 1).
- The incision is performed vertically (3-4 cm long), just distal to the inguinal ligament and lateral to the femoral artery.
- Subcutaneous tissue is dissected and the anterior fascia is sectioned vertically.
- Electrical stimulation (bipolar, 1 mA, 5 Hz, pulse width 100 $\mu$s) is used to identify individual muscle nerve motor branches based on their EMG response. Sensory branches are identified based on the absence of muscle response at stimulation with low intensity. In order to facilitate this mapping, colored surgical loops are used to tag nerve branches (e.g. green for branches to be lesioned; white for branches to be preserved). The use of surgical loops also allows for the atraumatic manipulation of nerve branches. Branches for rectus femoris and vastus intermedius are generally targeted because of their predominant involvement in spastic stiff knee gait [3, 9].
- Once functional mapping of nerves branches has been done, selective neurotomy is performed under surgical microscope. The nerve branch is isolated and the epineurium is opened with microscissors and individual fascicles are separated from each other. Electrical tripolar stimulation of individual groups of fascicles is performed to localize the motor response. Fascicles with the highest motor response (reflecting highest excitability) are preferentially sectioned in accordance with the preoperative chart. They are then resected over a few millimetres using microscissors. Distal and proximal stumps are coagulated using bipolar forceps to prevent axonal regeneration.
- In order to quantify the effect of neurotomy, the EMG responses to stimulation distal and proximal to the section of the nerve are compared. Distal stimulation shows the original nerve response since all fascicles are stimulated. Proximal stimulation shows the post-neurotomy response as it includes intact remaining fascicles only. If section is sufficient, a significant decrease in EMG response should occur from distal to proximal stimulation.
- The neurotomy procedure is repeated for each motor branch that has to be targeted in accordance with the preoperative chart.

Existing studies suggest that SFN durably improves objective gait parameters [1, 10]. This aligns with the findings of a recent systematic review, which concluded that selective neurotomy effectively reduces lower limb spasticity without negatively impacting walking speed [6].

## Indications

Selective Femoral Neurotomy (SFN) can be proposed to patients with focal quadriceps spasticity. In walking patients, this manifests typically as a *stiff knee gait*, a walking disorder characterized by reduced knee flexion during the swing phase of gait [1, 2, 10]. It can lead to costly compensatory movements, such as ipsilateral hip circumduction or contralateral vaulting, thus increasing energy expenditure during gait. In non-walking patients, focal quadriceps spasticity can induce debilitating spasms or nursing care difficulty. The goal of SFN is to limit such negative effects of spasticity in order to improve activities of daily living (e.g. going upstairs, tying shoes, squatting) and reduce the risk of fall.

SFN can be proposed regardless of the origin of spasticity (e.g., cerebral palsy, stroke, brain or spinal cord injury, multiple sclerosis), usually as a surgical long-lasting alternative to repeated botulinum toxin injections [1, 8]. Currently, the main indication is patients for whom quadriceps botulinum toxin injections have become less effective/tolerated.

Patient selection is performed by a multidisciplinary team including at least a neurosurgeon and a Physical Medicine and Rehabilitation physician. It requires a positive motor nerve block test with local anesthesia in order to (1) distinguish spasticity from contractures, (2) estimate the expected gait improvement, and (3) verify that upright posture is not compromised and that there is no decrease in voluntary knee extension velocity. This preoperative evaluation is ideally performed using quantitative gait analysis in walking patients.

Finally, for SFN to be performed, patients must accept to follow a rigorous postoperative rehabilitation program which includes walking rehabilitation.

## Limitations

- There is large inter-individual variability in the branching pattern of the femoral nerve [5]. Thus, the surgeon has to adjust to the anatomical specificities of the patient.
- When quadriceps spasticity has been present for a long time, it can be associated with muscle contractures and tendon retractions inducing knee deformity. This will not resolve with SFN alone and may require adjuvant orthopedic surgery. The preoperative femoral nerve block-test can help teasing apart spasticity – that would justify SFN – from associated neuro-orthopedic disturbances.
- In some cases, quadriceps spasticity can be associated with hamstring spasticity. This may require adjuvant hamstring neurotomy and/or orthopedic surgery. It highlights the importance of assessing the couple agonist/antagonist muscles in order to offer the best therapeutic strategy for each patient.

## How to avoid complications

 

- Nerve structures should be manipulated with care – using atraumatic surgical loops and holding only the epineurium – to avoid fascicle sideration.
- Precise functional mapping of nerve branches with combined electrical stimulation and EMG is mandatory as anatomical features alone are not reliable enough to identify motor branches.
- In order to preserve muscle strength and upright posture, only the most relevant nerve branches to the spastic heads of the quadriceps muscle are targeted to a level that generally does not exceed three-quarters of the section.

## Specific information for the patient

When discussing the benefits of the intervention, surgeons should insist on the following:

- Early postoperative intensive rehabilitation focusing on gait training is paramount for sustained clinical improvements.
- The Goal Attainment Scaling (GAS) method [4] can be used to establish *a priori* individualized objectives that can be used to evaluate intervention outcomes from the perspective of the patient (Table 1).

**Table 1:** Example Goal Attainment Scaling for Selective Femoral Neurotomy

Goal	Outcome rating
Increase walking perimeter	-2: Walking 600 m or less.
	-1: Walking 800 m.
	0: Walking 1000 m.
	+1: Walking 1200 m.
	+2: Walking 1400 m or more.
Facilitate the use of stairs	-2: Taking the stairs putting both feet on the same step and holding the handrail, for half a floor.
	-1: Taking the stairs putting one foot on each step and holding the handrail, for half a floor.
	0: Taking the stairs putting one foot on each step and holding the handrail, for one floor.
	+1: Taking the stairs putting one foot on each step and holding the handrail, for two floors or more.
	+2: Taking the stairs putting one foot on each step without holding the handrail, for one floor or more.

Scoring system: “-2” is the baseline (presurgery) level; “-1” represents a partial progression towards the goal; “0” is the expected level after surgery (most likely outcome); “+1” represents a better improvement than expected; “+2” is the best possible outcome that could be expected

When discussing intervention risks, surgeons should insist on the following:

- Standard surgical risks exist, such as thromboembolic complications and wound infection.
- A loss of knee locking may occur due to a decrease in the strength of quadriceps muscle but it is often temporary until the regrowth of the remaining motor fiber in the muscle (i.e. motor axonal spouting). Sensory disturbances and neuropathic pain may occur due to the manipulation of sensory nerve branches. These complications are rare and often temporary.
- Recurrence of spasticity can occur – especially in case of insufficient lesioning and in the absence of appropriate postoperative rehabilitation – but is rare (no more than 1% at 1 year) in our series [8].

## Key points summary

 

- An extended multidisciplinary clinical assessment inclu-ding motor nerve block test with quantitative motion analysis should be performed to (1) establish eligibility to SFN, and (2) determine the percentage of nerve to be lesioned (describe in the preoperative chart).
- Nerve mapping using electrical stimulation combined with EMG recording is mandatory during SFN.
- In order to not compromise the upright posture, usually a maximum of 75% section is performed.
- Postoperative rehabilitation is necessary for sustained clinical improvements, especially in walking patients.

## Supplementary Information

Below is the link to the electronic supplementary material.

Supplementary file 1 (mp4 403243 KB)

## Data Availability

No datasets were generated or analysed during the current study.
